# Mesenchymal stem cell therapy in type 2 diabetes mellitus

**DOI:** 10.1186/s13098-017-0233-1

**Published:** 2017-05-15

**Authors:** Li Zang, Haojie Hao, Jiejie Liu, Yijun Li, Weidong Han, Yiming Mu

**Affiliations:** 1 Department of Endocrinology, Chinese PLA General Hospital, Beijing, 100853 China; 2 Department of Molecular Biology, Institute of Basic Medicine, College of Life Science, Chinese PLA General Hospital, Beijing, 100853 China

**Keywords:** Mesenchymal stem cells, Type 2 diabetes mellitus, Insulin resistance

## Abstract

Type 2 diabetes mellitus (T2DM), which is characterized by the combination of relative insulin deficiency and insulin resistance, cannot be reversed with existing therapeutic strategies. Transplantation of insulin-producing cells (IPCs) was once thought to be the most promising strategy for treating diabetes, but the pace from the laboratory to clinical application has been obstructed due to its drawbacks. Mesenchymal stem cells (MSCs) harbor differentiation potential, immunosuppressive properties, and anti-inflammatory effects, and they are considered an ideal candidate cell type for treatment of DM. MSC-related research has demonstrated exciting therapeutic effects in glycemic control both in vivo and in vitro, and these results now have been translated into clinical practice. However, some critical potential problems have emerged from current clinical trials. Multi-center, large-scale, double-blind, and placebo-controlled studies with strict supervision are required before MSC transplantation can become a routine therapeutic approach for T2DM. We briefly review the molecular mechanism of MSC treatment for T2DM as well as the merits and drawbacks identified in current clinical trials.

## Background

Over recent decades, diabetes mellitus (DM) has become one of the major public healthcare problems worldwide [1]. It is estimated that 415 million adults have diabetes worldwide, and a further 318 million adults are estimated to have impaired glucose tolerance, and thus, be at high risk of developing diabetes in the future [2]. DM is a major risk factor for ischemic heart disease and stroke, which collectively account for high rates of morbidity and mortality among adult patients [3]. In addition, DM is the most common underlying cause of chronic kidney disease and blindness among adults [4, 5]. Improvement in glycemic control is the key to prevention of complications of DM. Type 2 diabetes mellitus (T2DM), which accounts for 90–95% of all DM cases, results from a combination of insulin resistance and dysfunction of insulin-producing pancreatic beta cells [6]. Initial treatment of T2DM generally includes oral anti-diabetic drugs; however, insulin is eventually needed for optimal glycemic control as the disease progresses. Although the currently available therapeutic regimens can ameliorate hyperglycemia or temporarily improve insulin sensitivity in target tissues, these can neither reverse insulin resistance nor the progressive and inexorable beta cell dysfunction [7]; that is, none of these therapies modulate the course of the disease. Dipeptidyl peptidase-IV (DPP-IV) inhibitors and glucagon-like peptide-1 (GLP-1) receptor agonists have been shown to improve beta cell function in animals, but these findings have not been verified in humans [8, 9]. Strategies to ameliorate peripheral insulin resistance and simultaneously promote beta cell regeneration may be the ideal therapeutic options for T2DM.

Transplantation of islet cells obtained from cadaveric donors was first performed in 1999 [10]. The success of this strategy was indicated by the demonstration of increased insulin production, normal blood glucose levels, normal glycosylated hemoglobin levels (HbA_1c_), and decreased requirement of insulin. However, the shortage of organ donors, complications of immunosuppressive agents, and exhaustion of the transplanted cells were the major obstacles to the widespread application of this therapeutic strategy [11, 12]. The identification of stem cells that possess the potential to differentiate into insulin-producing cells (IPCs), improve pancreatic regeneration, and ameliorate insulin resistance offers an alternative to islet cell transplant.

Transplantation of IPCs was once considered to be the most promising treatment approach for DM. However, suitable sources of IPCs free of ethical conflicts and lacking immunogenicity or tumorigenicity have not been established. In addition, there were apprehensions about the clinical efficacy of this approach due to the potential for cell exhaustion in vivo [13–16]. All of these obstacles have slowed the pace with which this approach could have transitioned from the laboratory to the clinical setting. In recent years, mesenchymal stem cells (MSCs) derived from different adult tissues have attracted significant attention for the treatment of DM. MSCs are known to promote the regeneration of pancreatic islet beta cells, protect endogenous pancreatic islet beta cells from apoptosis, and ameliorate insulin resistance of peripheral tissues by providing a supportive niche microenvironment driven by the secretion of paracrine factors or the deposition of extracellular matrix [17–22]. Not surprisingly, MSCs are now being intensively investigated for their efficacy and safety in both animals and humans. MSCs from different sources are known to exhibit unique characteristics. Bone marrow-derived MSCs (BM-MSCs) have been extensively investigated for their potential with regard to immunomodulation and tissue repair as well as the generation of IPCs via various differentiation protocols [23–27]. Interestingly, umbilical cord-derived MSCs (UC-MSCs) express embryonic markers and endodermal lineage markers. UC-MSCs have higher similarity to embryonic stem cells (ESCs) than other commonly used MSCs such as BM-MSCs and also have superior potential to differentiate into IPCs [28]. The ease of procurement, low immunogenicity with allogeneic sources, painless procedures for donors, and capacity for IPC differentiation make UC-MSCs an appealing alternative source of stem cells for treating T2DM [29, 30]. In addition, adipose tissue, placenta, amnion, and dental pulp have also been reported as alternative sources for cell therapy in diabetes. These MSCs can be induced to differentiate into physiologically competent functional IPCs, which may provide a source of alternative islets for cell replacement therapy [31–35].

## Molecular mechanism of action of MSCs

### Differentiation into IPCs

**Fig. 1:**
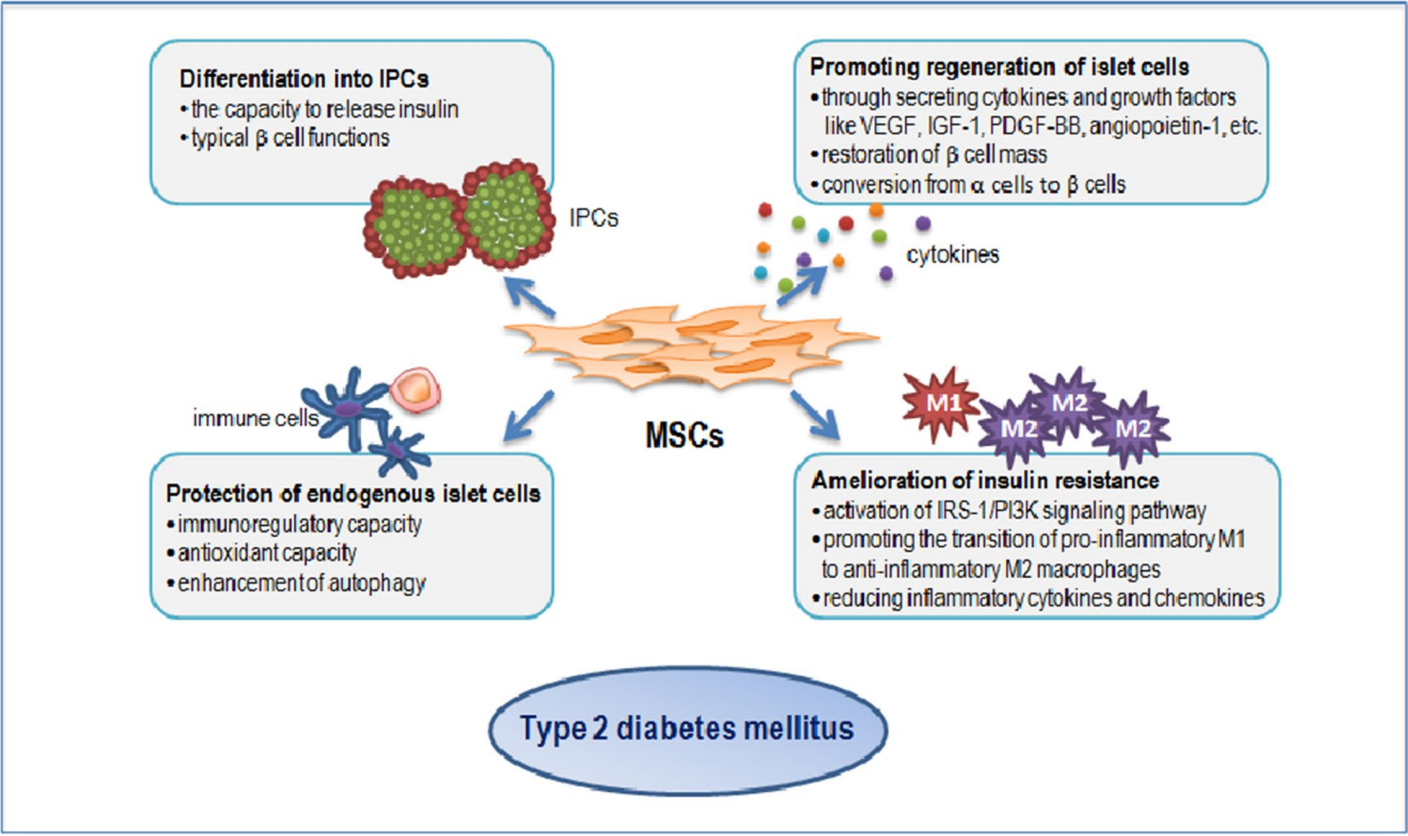
Diagram explaining the mechanism by which MSCs act on type 2 diabetes. MSCs exert beneficial effects on type 2 diabetes through differentiation into IPCs, promotion of islet cell regeneration, protection of endogenous islet cells and amelioration of insulin resistance. *IPCs* insulin-producing cells, *IGF*-*1* insulin-like growth factor-1, *VEGF* vascular endothelial growth factor, *PDGF* platelet-derived growth factor, *IRS*-*1* insulin receptor substrate-1, *PI3K* phosphoinositide 3-kinase

The potential to differentiate into IPCs was first considered to be the primary mechanism by which MSCs ameliorate hyperglycemia in T2DM (Fig. 1). Differentiation of the endocrine compartment of the pancreas is controlled by key transcription factors such as Pdx-1, Ngn-3, NeuroD1, Pax4, and Pax6 [36]. Correct reprogramming of cells to activate these pathways is necessary for inducing differentiation of MSCs into IPCs. Chen et al. first obtained incompletely differentiated IPCs expressing insulin and nestin by culturing rat BM-MSCs in serum-free medium in the presence of a high glucose concentration, nicotinamide, and β-mercaptoethanol [37]. Since then, modified protocols with different stimulating agents have been applied to improve the differentiation and efficacy; however, results in vivo have not been encouraging [38, 39]. Moriscot et al. first induced differentiation of human BM-MSCs into IPCs using adenoviral vectors coding for mouse Pdx-1 and Xie induced human BM-MSCs into IPCs using a three-step differentiation protocol (the addition of Activin A as the differentiating agent was the final step), and the resulting cells had the capacity to release insulin in a glucose-dependent manner [40, 41]. Chandra et al. [35] reported the generation of IPC aggregates from murine adipose tissue-derived stem cells, and these cells yielded numerous secretory granules within the cell cytoplasm after 10-day in vitro culture. Calcium alginate-encapsulated IPCs, when transplanted into streptozotocin-induced diabetic mice, restored normoglycemia within 2 weeks. Some researchers have recently indicated that UC-MSCs, especially Wharton’s jelly-derived MSCs (WJ-MSCs), which are easy to source and culture, can be successfully differentiated into IPCs. Some comparative studies have also demonstrated that WJ-MSCs have superior differentiation potential towards a mature beta cell phenotype as compared to BM-MSCs [42]. Chao et al. successfully differentiated WJ-MSCs into IPCs through a stepwise culture protocol using neuron-conditioned medium in vitro and proved that the differentiated IPCs exhibit typical beta cell functions in vivo [43]. Tsai et al. injected undifferentiated WJ-MSCs expressing green fluorescent protein (GFP) into non-obese diabetic (NOD) mice and observed co-localization of human C-peptide and GFP in the pancreas, indicating that WJ-MSCs differentiated into IPCs in vivo [44]. Wu et al. compared the differentiation potential of WJ-MSCs and BM-MSCs. The results showed that both stem cell types were able to form islet-like clusters in a medium containing nicotinamide, activin, hepatocyte growth factor, exendin-4, and pentagastrin. The expression of Pdx-1, insulin secretion, and mRNA expression of insulin and C-peptide in differentiated WJ-MSCs were greater relative to levels in differentiated BM-MSCs [42].

However, the short-term survival time of the differentiated cells, which resulted from the usage of stimulating agents and adenoviral vectors in the differentiation process limited their application. Direct transplantation of MSCs was once thought to be the most effective way to avoid this unwanted consequence. In fact, transplanting undifferentiated human placenta-derived MSCs or biocompatible macrocapsules with differentiated IPCs under the kidney capsules of STZ-induced diabetic mice, both resulted in a reduction of hyperglycemia and restoration of normoglycemia 15 days post transplantation [34]. Transplanted MSCs were preferentially located in the damaged pancreatic tissue of diabetic mouse models. However, as only a small fraction of donor insulin-positive cells was found in the pancreas, they could not completely account for the renewal of the islet cells [13]. Ianus et al. observed significant regeneration of adult beta cells in diabetic mice after transplantation of BM-MSCs, despite only 1.7–3% of islet beta cells being of bone marrow origin [14]. Lechner et al. found no significant trans-differentiation of BM-MSCs into pancreatic beta cells in vivo (among >100,000 beta cells, only two beta cells were potentially from donors) [15]. Choi et al. reported that the GFP-labeled cells were found in the islets after bone marrow transplantation, but none of these cells expressed insulin [16]. This information led to the thought that the differentiated islet progenitors were not the source of the regenerated pancreatic beta cells. Whether the restoration of euglycemia was due to MSC differentiation still remains controversial.

### Promoting the regeneration of pancreatic islet beta cells

In addition to the capacity to differentiate into IPCs, MSCs also promote the regeneration of endogenous pancreatic islet beta cells by migrating to the injured islet cells. The MSCs participate in the repair processes by secreting a variety of cytokines and growth factors that have both paracrine and autocrine activities [17]. Significant endogenous beta-cell regeneration and islet architecture restoration has been observed after single or multiple infusions of MSCs [18, 19]. This effect might have been mediated by the secretory effects of MSCs, as the conditioned medium from cultured MSCs had the same capacity to regulate blood glucose in diabetic mice [20]. Lee et al. found that MSCs migrated to the islets of streptozocin (STZ)-induced diabetic mice where they promoted tissue repair primarily by creating a microenvironment that allowed endogenous cells to proliferate and regain their normal function [21]. The paracrine factors, such as vascular endothelial growth factor (VEGF)-alpha, insulin-like growth factor (IGF)-1, platelet-derived growth factor (PDGF)-BB, and angiopoietin-1, also play an integral role in the process of cell regeneration [22].

In Fox-01 ablation mice, a number of dedifferentiated beta cells were reprogrammed into alpha cells, which resulted in insulinopenia with hyperglucagonemia in early T2DM [45]. Another study provided circumstantial evidence that the phenomenon of beta-cell reprogramming into alpha cells occurs in humans [46]. In the mouse model of acute pancreatitis with severe defects in beta cells, islet alpha-cells converted directly into beta cells to compensate for their absence, which resulted in the restoration of beta-cell function [47]. All these results indicated that islet alpha cells have an inherent potential for spontaneous reprogramming into beta cells. When Arx is aberrantly expressed in mature beta cells, conversion of beta cells into glucagon-producing cells occurs in adult mice [48]. Ectopic expression of Pax4 can force mature endocrine alpha cells to function like beta cells and reverse the consequences of STZ-mediated DM [49]. However, such a spontaneous regeneration response was observed only in the case of extreme injury or beta-cell ablation, which is usually not the case in humans. Our unpublished studies demonstrated that MSC infusion was sufficient to promote trans-differentiation of alpha cells into beta cells. In mice with STZ-induced T2DM, intravenous infusion of allogeneic BM-MSCs resulted in the appearance of considerable insulin/glucagon double hormone-positive cells followed by restoration of beta-cell mass and dramatic amelioration of hyperglycemia. The intriguing conversion from alpha cell to beta cell can provide a revolutionary paradigm for treating DM via MSC infusion.

### Protection of endogenous pancreatic islet beta cells

In addition to their regenerative properties, MSCs have also demonstrated an immunoregulatory capacity. MSCs are also known as immunoprivileged cells because of the low intracellular expression of class II major histocompatibility (MHC) proteins and co-stimulatory molecules [50, 51]. As the main effectors of the adaptive immune response, T lymphocytes play a prominent role in autoimmune disease and transplant rejection. Some studies have demonstrated that MSCs suppress the proliferation of T lymphocyte by inhibiting the energy metabolism of the T cell population, promoting T-cell tolerance, or by inducing proliferation of regulatory T-cell populations [52]. Moreover, MSCs also inhibited the proliferation of B cells and stopped a variety of immune cell functions, including cytokine secretion and cytotoxicity of T and natural killer (NK) cells, B cell maturation [53], and antibody secretion. The immunosuppressive effects of MSCs attenuated the autoimmune processes that lead to the destruction of pancreatic beta cells.

MSCs have been reported to promote islet survival against hypoxia and oxidative stress [54]. In the study by Chdravanshi et al. [54], after 48 h of direct contact co-culture with Wharton’s jelly-derived MSCs, islet cells exhibited higher viability and reduced apoptosis as compared with their controls without co-culture. In addition to amplified expression of anti-inflammatory cytokines like TGF-β and TNF-α and lower levels of pro-inflammatory cytokines, co-cultured islet cells revealed reduced levels of reactive oxygen species, nitric oxide, and super oxide ions, suggesting the protective effect of MSCs on islet cells against oxidative stress-mediated cellular injuries. Given that oxidative stress injury induced by hyperglycemia is recognized as a major etiological factor in the development of diabetes, further investigation of the antioxidant capacity of MSCs for the promotion of islet survival may validate the utility of MSC co-transplantation with islet transplantation. Autophagy is a common intracellular degradation process by which eukaryotes maintain intracellular homoeostasis via degradation and recycling of damaged organelles and toxic proteins [55]. Autophagy plays an indispensable role in a variety of diseases, such as neurodegenerative disorders and cardiovascular diseases. Basal autophagy is essential for maintaining the architecture and function of pancreatic islet beta cells. Both the deficiency and enhancement of autophagy play a role in the pathogenesis of T2DM [56–59]. In a recent study, Zhao et al. found that co-culture with BM-MSCs significantly alleviated the glucotoxicity of INS-1 cells that had been induced by prolonged exposure to high glucose. Glucose toxicity in INS-1 cells was characterized by decreased cell viability, increased cell apoptosis, and impaired basal insulin secretion and glucose-stimulated insulin secretion. A later study showed that the protective effect of BM-MSCs on the INS-1 cells was mediated by promotion of autophagosome and autolysosome formation, which was considered to be a symbol of autophagy [60]. Another study indicated that UC-MSCs improve wound healing in DM by inducing autophagy [61]. All these results provide evidence supporting enhancement of autophagy by MSCs as an ideal strategy for the treatment of T2DM.

### Amelioration of insulin resistance

Dysfunction of insulin-producing pancreatic islet beta cells and insulin resistance co-exist in T2DM. Therefore, the mechanism underlying MSC treatment of DM in the experiments described above could not be adequately explained solely by the potential ability of MSCs to promote pancreatic islet beta-cell function. Si et al. for the first time found that BM-MSC transplantation alleviated hyperglycemia in rats with high-fat diet/STZ-induced T2DM by activating the insulin receptor substrate (IRS)-1 signaling pathway. This resulted in increased translocation and expression of GLUT-4, which further resulted in BM-MSC–mediated amelioration of insulin resistance in peripheral insulin target tissues [18]. Interestingly, infusion of MSCs during the early phase (7 days) could restore β-cell function, ameliorate the destruction of pancreatic islets, promote recruitment of MSCs to the damaged tissues, and reduce insulin resistance, whereas infusion in the late phase (21 days) merely ameliorated insulin resistance, suggesting a reasonable therapeutic time window in the early phase of diabetes [18]. Following this intriguing finding, Hughey et al. had a serendipitous discovery that glucose uptake in peripheral tissues, including skeletal muscle and adipose tissue, was elevated in MSC-treated mice with myocardial infarction. Furthermore, enhanced glucose uptake in these tissues was associated with improved insulin signaling as assessed by Akt phosphorylation and the expression of GLUT-4 [62]. However, the mechanism by which MSCs ameliorated insulin resistance could not be understood completely.

Insulin resistance is now considered to be closely related to systemic chronic low-grade inflammation. Cytokines and chemokines, such as tumor necrosis factor alpha (TNF-α) and interleukin-1 beta (IL-1β), produced by adipose tissue macrophages (ATMs) in an M1 pro-inflammatory state have been identified as crucial effectors in the initiation of inflammation and the development [63] of insulin resistance. However, an anti-inflammatory subset called M2, or alternatively activated macrophages, have been shown to play a role in preventing insulin resistance [64]. MSCs have been shown to exert anti-inflammatory effects by promoting M2 polarization in skin wounds and rhabdomyolysis-induced kidney injury, both in vitro and in vivo [65, 66]. Our research team recently confirmed that UC-MSCs alleviate insulin resistance in T2DM rats by reprogramming classically activated macrophages (M1, pro-inflammatory) into an alternatively activated phenotype (M2, anti-inflammatory). Further analysis showed that M1-stimulated UC-MSCs increased expression of IL-6. IL-6 upregulated IL4R expression, promoted phosphorylation of STAT6 in macrophages, and ultimately reprogrammed macrophages into an M2 phenotype [67]. In addition, conditioned media from adipose tissue-derived MSCs reversed insulin resistance in insulin-resistant cell models, as evidenced by restored insulin and stimulated glucose uptake, via up-regulation of the GLUT4 gene and reductions in IL-6 and plasminogen activator inhibitor-1 (PAI-1) gene expression [68]. Other results showed that UC-MSCs alleviate insulin resistance in rats with T2DM by regulating the expression of NLRP3 inflammasome in peripheral insulin target tissues. Although many questions about MSCs and insulin resistance remain unanswered, these results shed new light on the effects of autologous MSCs on obesity-related insulin resistance in T2DM.

## Clinical application of MSC transplantation for the treatment of T2DM

In animal models, MSC treatment demonstrated exciting therapeutic effects on glycemic control by restoring islet function and ameliorating insulin resistance. These results have now been translated into clinical practice. A total of 96 registered phase I/II clinical studies among T2DM patients can be found with the clinical trials registry (http://www.clinicaltrials.gov). Thirteen papers evaluating the clinical effects of MSC treatment in the management of T2DM have been published, although these include only four randomized, placebo-controlled studies (Table 1).

**Table 1:** Summary of MSC-based therapies for type 2 diabetes

No	Stem cell type	Randomized placebo-control study	Mean dose of injected cells/kg	Mode of injection	Follow-up period	Assessment of beta-cell function	Assessment of insulin sensitivity	Publication
1.	MNCs	–	–	Intrapancreatic	12 month	Fasting C-peptide		Estrada et al. [69]
2.	BM-MNCs	–	3.1 × 10^6^	Intra-pancreatic	6 months	Fasting C-peptide, glucagon stimulated c-peptide, HOMA-β	HOMA-IR	Bhansali et al. [70]
3.	BM-MNCs	Yes	3.2 × 10^8^	Intra-pancreatic	12 months	Fasting C-peptide, glucagon stimulated c-peptide, HOMA-β	HOMA-IR	Bhansali et al. [71]
4.	UC-MSCs	–	1 × 10^6^	IV + intrapancreatic on day 5	12 months	Fasting C-peptide and HOMA-β	HOMA-IR	Liu et al. [72]
4.	BM-MNCs	Yes	2.8 × 10^9^	Intra-pancreatic	33 months	Mixed meal tolerance test		Hu et al. [73]
6.	MNCs	–	(5–7) × 10^8^	IV or pancreatic arterial infusion	6 months	Fasting C-peptide, glucagon stimulated c-peptide, HOMA-β	HOMA-IR	Sood et al. [75]
7.	PD-MSCs	–	1.35 × 10^6^	IV	6 months	Fasting C-peptide	Fasting insulin	Jiang et al. [77]
8.	MSCs	–	1.8 × 10^6^	IV	6 months	Fasting C-peptide		Kong et al. [78]
9.	BM-MSCs	–	(0.3–2.0) × 10^6^	IV	24 months	Fasting C-peptide	Fasting insulin	Skyler et al. [83]
10.	BM-MNCs	Yes	3.8 × 10^9^	Pancreatic arterial infusion	12 months	Fasting C-peptide and OGTT	HOMA-IR	Wu et al. [85]
11.	BM-MNCs	–	3.76 × 10^8^	Pancreatic arterial infusion	720 days	Mixed meal tolerance test		Wang et al. [86]
12.	CB-SC	–	–	IV	12 months	Fasting C-peptide and HOMA-β	HOMA-IR	Zhao et al. [87]
13.	BM-MSCs and MNCs	Yes	MSCs: 1 × 10^6^/kg; MNCs:10^9^ in total	Intra-pancreatic	12 months	Fasting C-peptide, glucagon stimulated c-peptide, HOMA- β, hyperglycemic clamp	HOMA-IR, HOMA-S and insulin sensitivity index during hyperglycemic clamp	Bhansali et al. [88]

*MSCs* mesenchymal stem cells, *MNCs* mononuclear stem cells, *UC* umbilical cord, *PD* placenta-derived, *CB*-*SC* cord blood-derived multipotent stem cells, *IV* intravenous, *HOMA*-*β* homeostatic model assessment of beta cell function, *HOMA*-*IR* homeostasis model assessment of insulin resistance

### Clinical efficacy

As the clinical efficacy of MSC treatment for T2DM is a major concern, HbA_1c_ reduction and insulin requirements were frequently used as measures to assess the efficacy of MSC treatment for T2DM. In 2008, Estrada et al. [69] first found that combination therapy with BM-MSC transplantation and hyperbaric oxygen therapy (HOT) effectively reduced HbA_1c_ levels in patients with T2DM for up to 1 year. In 2009, Bhansali et al. [70] demonstrated that the insulin requirements of 7 out of 10 patients decreased by 75%, and three patients were able to discontinue insulin after a single BM-MSC transplantation. Bhansali et al. [71] reported that 9 out of 11 patients (82%) achieved the primary end point of a 66.7% decrease in their insulin requirement. Together, these results proved that MSC transplantation should be evaluated further for treatment of T2DM. However, the question about the duration of the effectiveness of MSC treatment was raised simultaneously. Wang et al. found that HbA_1c_ levels decreased transiently as early as 1 month; however, HbA_1c_ levels did not decline continuously until the subsequent follow-up. The same phenomenon was also observed in two other clinical trials carried out by Liu et al. and Hu et al.; HbA_1c_ decreased in first 3 months and 1 year, but gradually increased in the next 9 months and 2 years, respectively [72, 73]. Currently, this is a matter of concern for the development of use of MSCs in the management of T2DM.

Whether this poor response is due to route of administration of MSCs remains to be ascertained. On the other hand, more studies have proved that biological activity factors, such as VEGF, IGF-1, and β-FGF, secreted by MSCs can regulate the local microenvironment of the damaged tissue, inhibit cell apoptosis, improve the immune defense system, and promote tissue regeneration and revascularization. Systemic infusion of MSCs was believed to be superior to local injection because the therapeutic effects of MSCs were mainly derived from their secretory effects rather than their differentiation effect. Furthermore, systemic infusion not only reduces trauma but is also more convenient, especially for patients requiring repeated administration. Although solid evidence from a rat model of diabetes suggested selective localization of BrdU-labeled MSCs into the pancreas after MSC infusion through the tail vein [74], how the route of administration affects the clinical efficacy of MSCs remains unclear. To address this question, Sood et al. infused ^18^F-FDG–labeled BM-MSCs via either the peripheral intravenous route or targeted routes into the superior pancreaticoduodenal artery and splenic artery. Positron emission tomography (PET) was used to track the homing and retention of the MSCs. The results showed that the best homing of MSCs in the pancreas was observed when cells were infused in the superior pancreaticoduodenal artery. No discernible homing of MSCs was observed in the intravenous route group; cells infused via the intravenous route first entered in the lung fields and then migrated and gained access to the systemic circulation. They also found that the clinical efficacy (HbA_1c_ reduction and insulin requirement) of cells delivered via the intravenous route was inferior to that of cells delivered via the pancreaticoduodenal artery and the splenic artery [75]. Thus, the authors concluded that the intravenous route was the least effective route for stem cell infusion. As the study had a small sample size, this conclusion needs to be verified in larger scale investigations.

The next question about single versus multiple injections also needed consideration. A few animal studies have compared the therapeutic effects of multiple MSC injections versus a single injection on diabetes [18, 19, 76]. The results reveal that the beneficial effect of a single infusion of MSCs for ameliorating hyperglycemia is perhaps maintained only for a period not exceeding 4 weeks [18]. However, currently there is no consensus on the optimal time or interval of MSC infusion due to a lack of direct comparisons of single versus multiple injections in diabetes patients. Considering a single administration of MSCs may not be enough to maintain a therapeutic effect over a long period of time, a number of clinical studies have been carried out using multiple MSC injections, typically 2–4 times with 2- to 12-week intervals [71, 77–79]. Bhansali et al. administered a second injection of MSCs via the antecubital vein 12 weeks after the first injection via the transfemoral route into the superior pancreaticoduodenal artery. This resulted in a further reduction of insulin dose requirements [71], suggesting that multiple injections of stem cells may produce a more durable effect. Further research is needed to explore the cumulative therapeutic effect of multiple MSC injections with short intervals.

The dosage of MSC infusions is another uncertain issue. Various factors may influence the selection of the treatment dose, such as the type of MSCs, route of cell delivery, viability and purity of MSCs, and condition of the patient [80]. In current clinical studies, the mean dose of injected cells ranges from 1 × 10^6^ to 2.6 × 10^7^/kg of bodyweight due to the use of different cell types and counting methods [81]. Dose-dependent therapeutic effects of MSC infusion have been reported in animal experiments [82]. Similarly, a direct correlation was found between the number of allogeneic MSCs applied and the therapeutic outcomes in treating type 2 diabetes. In a randomized, placebo-controlled, dose-escalation study, patients with type 2 diabetes inadequately controlled with oral antidiabetic agents received allogeneic BM-MSCs at a dose of 0.3 × 10^6^, 1.0 × 10^6^, or 2.0 × 10^6^/kg. At week 12, the target HbA_1c_ <7 mg/dL was achieved by 33% of the patients who received the 2.0 × 10^6^/kg dose and 13.3% of those who received the 0.3 × 10^6^/kg dose [83]. With only scattered evidence showing the benefits of a high dosage of MSCs, further research is still needed to determine an appropriate cell dose that is most effective but still safe in a well-designed, dose-escalating clinical trial.

HOT can promote stem cell mobilization and endothelial progenitor cell release by increasing the concentration of carbon monoxide synthase [84]. It was hypothesized that the combination of HOT and MSC transplantation can have a synergistic effect. Estrada et al. and Wang et al. proved that the combined strategy of HOT and MSCs led to reductions in HbA_1c_ levels and insulin requirement [69], while no interaction between HOT and BM-MSC infusion was observed by Wu et al. [85].

However, until now, there had been no studies comparing the clinical efficacy of different sources of MSCs, possibly due to the lack of universally accepted criterion for defining MSC phenotypes and functional properties.

### Evaluation of islet function

Improvement in islet function was regarded as the primary mechanism of action of MSCs in the treatment of T2DM. Fasting levels of C-peptide, glucagon-stimulated C-peptide, or C-peptide area under the curve (AUC_C-pep_), and homeostatic model assessment of beta-cell function (HOMA-β) have been used as evaluation indices in various clinical studies. As fasting C-peptide is the most convenient and effective indicator, it was evaluated in all 13 published clinical studies [69–73, 75, 77, 78, 83–87] and the results obtained have been encouraging. The functional capacity of islet cells in early phase insulin secretion and reservation were also assessed. Glucagon-stimulated C-peptide was found to be significantly increased in MSC-treated cases compared with controls [71], and the area under the curve for C-peptide (AUC_C-pep_) increased 43.8% from baseline following MSC implantation [85]. In contrast to these favorable outcomes, Skyler et al. and Kong et al. found that an increase in fasting C-peptide levels was not detectable after transvenous MSC transplantation [78, 83]. Wang et al. demonstrated that fasting C-peptide increased significantly from baseline at 3 months after intrapancreatic MSC transfusion, but was similar to baseline levels at later time points [78, 83]. Similar results were also observed in clinical studies by Liu et al. and Hu et al. [72, 73]. Liu et al. found that fasting C-peptide progressively increased with the peak value achieved at 6 months, and a slight decrease later at 12 months [72]. Hu et al. also reported an improvement in C-peptide levels at 1 year after MSC implantation, but later there was a decreasing trend in C-peptide levels over the following 2 years [73]. Longer disease duration, older age, poor islet function and other complications of T2DM were associated with a less conducive microenvironment for transplanted cells. Under these circumstances, there was only a short-term recovery of islet cell function and a negative impact on the long-term clinical efficacy of transplanted MSCs.

### Evaluation of insulin resistance

Unlike promoting islet function, amelioration of insulin resistance using MSC treatment had not been given adequate attention in either animal experiments or clinical trials. Homeostatic model assessment of insulin resistance (HOMA-IR) has been commonly used to evaluate the insulin resistance in peripheral tissues; however, glucose infusion rate (GIR) has been associated with implementation difficulties. Bhansali et al. found that intrapancreatic BM-MSC transplantation did not improve HOMA-IR results [70]. Significant improvement in HOMA-IR levels transplantation at 6 months, but not at 12 months was observed in a group receiving combined transvenous and intrapancreatic MSC [71]. Liu et al. showed that fasting serum C-peptide and 2-h postprandial C-peptide levels were decreased to different degrees 1 month after intrapancreatic WJ-MSC transplantation in subjects receiving insulin therapy, along with a reduction in their insulin requirements at the respective time points. The most probable explanation for this contradictory phenomenon was that infused WJ-MSCs rapidly improved general insulin resistance, leading to a reduction in endogenous insulin secretion and the need for exogenous insulin injection [50]. In a clinical trial carried out by Kong et al., fasting blood glucose (FBG) and postprandial blood glucose (PBG) of patients in the intervention group reduced significantly reduced after transvenous UC-MSC transfusion, whereas there was no significant increase in C-peptide levels. The results implied that UC-MSCs might have the capacity to restore insulin sensitivity in tissues to an extent that further corrected hyperglycemia [78]. Unfortunately, these two studies did not assess insulin resistance. As the experiments indicated that MSCs had the potential of ameliorating insulin resistance, which might have contributed to the long-term glycemic control of T2DM in vivo and in vitro, indices evaluating insulin resistance should be measured in future clinical trials to elucidate the fundamental mechanism of the effects of the MSCs.

Our recent results showed that insulin requirements decreased by 50% and GIR significantly improved by 6 months after multiple intravenous injections of UC-MSCs in T2DM patients with poor glycemic control. This result confirmed that UC-MSCs reduce hyperglycemia in T2DM patients in part by ameliorating insulin resistance of peripheral tissue.

### Adverse events

Patient safety and the risk to benefit ratio continue to be the most important considerations in clinical practice Therefore, adverse events were monitored in all of the clinical trials evaluating MSC treatment for T2DM. Potential risks of MSC treatment included pulmonary and upper respiratory adverse events (intravenous injection that leads to infused cells passing through the lungs), acute allergic and immunologic adverse events, and damage caused by puncture and unwanted tissue formation. No acute allergic and immunologic adverse events were reported in all 13 studies [69–73, 75, 77, 78, 83–87] Unwanted tissue formation also was not found, but needs to be assessed over long-term follow-up. A low incidence of punctural hemorrhage, posttraumatic pain, and subcutaneous hematoma at the injection site following intrapancreatic MSC transplantation was reported by Wu et al., Liu et al., and Bhansali et al., respectively [70, 72, 85]. Mild and moderate fever with spontaneous remission after transvenous MSC transplantation was reported in 13.6–22.2% of patients [72, 78]. Transient self-limiting nausea, vomiting, headache, abdominal pain, and upper respiratory tract infection occasionally occurred after the MSC transplantation [70, 72, 83]. Minor hypoglycemia resulting from the use of anti-diabetic drugs was common, whereas severe hypoglycemia was not frequently reported as the patients were closely monitored by the sponsors in the follow-up period.

## Conclusions

Results available from animal and human studies are encouraging, and MSC therapy may represent a new paradigm for optimizing glycemic control in T2DM. However, many issues remain unresolved. Animal experiments demonstrated that MSCs relieve hyperglycemia by differentiating into IPCs, improving pancreatic regeneration, promoting the conversion of alpha cells to beta cells, and ameliorating insulin resistance. As the animal models used for such experiments do not truly represent T2DM patients, the fundamental mechanisms involved in the improvement in beta cell function and/or insulin sensitivity require further investigation. To make new cell therapy-based strategies a clinical reality, it is fundamentally necessary to identify sources of MSCs that do not have any ethical conflicts. Maximal efficacy and durable therapeutic effects along with minimal side effects are preferable for a particular therapeutic application. Duration of DM, residual beta-cell function, and the severity of complications (which reflect the microenvironment conditions) will affect treatment efficacy. Thus, the method of selection of appropriate patients for this treatment needs to be determined. In addition, the ideal route of administration of stem cells, whether targeted or peripheral, should be identified. In the targeted approach, determining the optimal administration method, whether via the dorsal pancreatic artery, superior pancreaticoduodenal artery, or splenic artery, is also important, as the targeted approach may be associated with better outcome than simple peripheral administration of stem cells. However, aspects like how long engrafted MSCs survive in vivo and maintain their functions, the optimal dosing regimen, and the long-lasting effect of multiple injections need further evaluation.

Importantly, MSCs have the potential for malignant transformation due to their multipotent or pluripotent features; therefore, patients undergoing this type of treatment should be closely monitored for the development of any neoplasia. Thus, multi-center, large-scale, double-blind, and placebo-controlled studies with strict supervision are required before MSC transplantation becomes a routine therapeutic approach for T2DM.

## Abbreviations

T2DM: type 2 diabetes mellitus
IPCs: insulin-producing cells
MSCs: mesenchymal stem cells
DM: diabetes mellitus
DPP-IV: dipeptidyl peptidase-IV
ESCs: embryonic stem cells
MHC: major histocompatibility
PET: positron emission tomography
GIR: glucose infusion rate
FBG: fasting blood glucose
